# Cannabidiol markedly alleviates skin and liver fibrosis

**DOI:** 10.3389/fphar.2022.981817

**Published:** 2022-10-19

**Authors:** Carmen del Río, Francisco Ruiz-Pino, María E. Prados, Bernd L. Fiebich, Manuel Tena-Sempere, Eduardo Muñoz

**Affiliations:** ^1^ Instituto Maimónides de Investigación Biomédica de Córdoba-IMIBIC, Cordoba, Spain; ^2^ Departamento de Biología Celular, Fisiología e Inmunología, Universidad de Córdoba, Cordoba, Spain; ^3^ Hospital Universitario Reina Sofía, Cordoba, Spain; ^4^ VivaCell Biotechnology España, Cordoba, Spain; ^5^ VivaCell Biotechnology GmbH, Dezlingen, Germany; ^6^ CIBER Fisiopatologia de la Obesidad y Nutrición, Instituto de Salud Carlos III, Cordoba, Spain

**Keywords:** cannabidiol, fibrosis, systemic sclerosis, non-alcoholic fatty liver disease, COL1A2

## Abstract

Cannabidiol (CBD) has been suggested as a potential therapy for inflammatory and fibrotic diseases. Cannabidiol was demonstrated to reduce alcohol-induced liver inflammation and steatosis but its specific activity on the fibrotic process was not investigated. Herein, the antifibrotic effects of cannabidiol in the skin were analysed *in vitro* using NIH-3T3 fibroblasts and human dermal fibroblasts and *in vivo* using the bleomycin-induced model of skin fibrosis. In a second model, non-alcoholic liver fibrosis was induced in mice by CCl_4_ exposure. Cannabidiol was administered daily, intraperitoneally in mice challenged with bleomycin and orally in CCl_4_ mice, and skin and liver fibrosis and inflammation were assessed by immunochemistry. Cannabidiol inhibited collagen gene transcription and synthesis and prevented TGFβ-and IL-4 induced fibroblast migration. In the bleomycin model, cannabidiol prevented skin fibrosis and collagen accumulation around skin blood vessels, and in the CCl_4_ model cannabidiol significantly attenuated liver fibrosis measured by picrosirius red and Tenascin C staining and reduced T cell and macrophage infiltration. Altogether, our data further support the rationale of the medicinal use of this cannabinoid, as well as cannabis preparations containing it, in the management of fibrotic diseases including Systemic Sclerosis and Non-Alcoholic Fatty Liver Disease.

## Introduction

Cannabidiol (CBD), the main non-psychotropic component of *Cannabis sativa* L. (Cannabaceae), has aroused much interest due to its broad range of therapeutic potential. CBD antiinflammatory and antifibrotic properties stem from multiple pharmacological mechanisms but the relative contribution of each pathway is not known. CBD shows a very low affinity to CB_1_ and CB_2_ receptors, and recent evidence raised the possibility that CBD can act as a negative allosteric modulator of CB_1_ receptor (Laprairie et al., 2015). Despite CBD behaves as a low affinity agonist, several studies support that CBD effects can be partially attributed to its activity on CB_2_ receptor (Martinez-Pinilla et al., 2017; Vilela et al., 2017). CB_1_ and CB_2_ receptors have been shown to play opposite roles in experimental models of fibrosis. While CB_1_ inactivation exerted antifibrotic effects by indirectly regulating leukocyte infiltration (Marquart et al., 2010) and the production of transforming growth factor beta 1 (TGFβ1) (Teixeira-Clerc et al., 2006), activation of CB_2_ receptors was reported to reduce tissue fibrosis in various rodent models of organ fibrosis, including skin (Akhmetshina et al., 2009), liver (Munoz-Luque et al., 2008) and heart (Li et al., 2016).

Apart from canonical cannabinoid receptors, CBD acts on numerous biological targets able to downregulate proinflammatory and profibrotic cytokines, including the modulation of different transient receptor potential vanilloid (TRPV) channels and GPR55 receptor antagonism (Sunda and Arowolo, 2020). In addition, CBD stimulation of PPARγ may inhibit the transcription of proinflammatory NF-κB dependent genes (Scirpo et al., 2015) and abrogate collagen synthesis by interfering TGFβ signalling (Ghosh et al., 2009). CBD also enhances adenosine signaling to reduce inflammation (Carrier et al., 2006), which has been shown to alleviate bleomycin-induced lung fibrosis (Chen et al., 2017). Moreover, the antioxidant properties of CBD can also contribute to its effects by downregulating intracellular ROS generation and lipid peroxidation (Sunda and Arowolo, 2020). More recently, it has been shown that the antioxidant activity of CBD can be also mediated by targeting Bach1 and inducing the expression of HMOX-1 (Casares et al., 2020).

CBD administration has been described to reduce inflammation and fibrosis in different experimental disease models, such as allergic asthma (Vuolo et al., 2019), diabetic cardiomyopathy (Rajesh et al., 2010) and alcohol-related fibrosis in the liver (De Ternay et al., 2019). Recently, we demonstrated that two different CBD aminoquinone derivatives, acting as dual PPARγ/CB_2_ ligand agonists, inhibit fibroblast differentiation and collagen deposition *in vitro* and alleviate inflammation and fibrogenesis in a mouse model of experimental systemic sclerosis, when delivered either intraperitoneally or orally (Del Rio et al., 2018; Del Rio et al., 2018; Garcia-Martin et al., 2018). Similarly, the (+)-enantiomer of CBD and its derivative (+)-CBD hydroxypentylester mitigated immune cell infiltration and renal fibrosis (Gonzalez-Mariscal et al., 2021). Randomized controlled trials have shown that CBD and CBD derivatives are well-tolerated in a wide range of disease conditions with limited side effects (Devinsky et al., 2018; Taylor et al., 2018; Taylor et al., 2019) and the FDA approval of a CBD oral solution (Epidiolex^®^) for certain types of epilepsy supports its medicinal use.

Fibrosis is a lifelong pathological condition characterized by the excessive collagen and extracellular matrix (ECM) accumulation. Fibrosis can be classified based on anatomical location in systemic or organ specific fibrosis, with increased frequency in the skin, liver, heart, kidneys, and lungs (Pryimak et al., 2021). The etiology of fibrotic disorders is heterogeneous and the causative mechanisms remain still elusive. Systemic sclerosis (SSc) is a highly heterogeneous immune-mediated rheumatic disease characterised by the presence of vasculopathy that precedes fibrosis in the skin and internal organs (Varga and Abraham, 2007). In addition, abnormal epigenetic modifications in fibroblasts, endothelial cells and immune cells also participate in pathogenic pathways of SSc (Tsou et al., 2021). Damaged endothelial cells release growth factors and cytokines that promote inflammatory infiltration and autoimmunity. The long-lasting synthesis of proinflammatory mediators by the infiltrated immune cells triggers fibroblast proliferation and differentiation. These cells become the primary source of extracellular matrix causing excessive remodelling and tissue dysfunction (Kendall and Feghali-Bostwick, 2014). In the same way, hepatic stellate cells activated upon injury are responsible of the scarring response of liver, which can result from toxic, metabolic, and viral insults. Liver disease progression by sustained inflammation and progressive fibrosis leads to cirrhosis, a major cause of morbidity and mortality worldwide (Hernandez-Gea and Friedman, 2011). At the moment, there are no effective antifibrotic treatments for human use. In this context, the strong antiinflammatory and antifibrotic potential of CBD represent a useful pharmacological approach for the treatment of fibrosis in both pathological conditions. Therefore, we have investigated the ability of CBD to ameliorate fibrosis in experimental models of SSc and non-alcoholic liver fibrosis.

## Methods

### Cell lines

NIH-3T3 and human dermal fibroblasts (NHDFs) were cultured in DMEM supplemented with 10% FBS, 2 mM l-glutamine and 1% penicillin/streptomycin. All cells were maintained at 37°C and 5% CO_2_ in a humidified atmosphere. 99.73% pure synthetic trans (−) Cannabidiol was obtained from Symrise AG, lot number 10300010 (Holzminden, Germany).

### Col1a2 and CAGA transcriptional assay and collagen synthesis measurement

NIH-3T3 cells were seeded in 24-well plates (5 × 10^4^ cells/well). After 24 h, cells were transiently transfected with Col1a2-luc (1 μg/well) using Roti©-Fect (Carl Roth, Karlsruhe, Germany) following manufacturer’s specifications. 24 h after transfection, cells were pre-treated with CBD for 1 h at the indicated concentrations and stimulated with TGFβ1 (5 ng/ml) for the following 24 h. Then, cells were lysed in 100 μL of lysis buffer and luciferase activity was measured using the Dual-Luciferase^®^ reporter assay system (Promega; Madison, WI, United States). Human dermal fibroblasts were seeded in 24-well plates (6 × 10^4^ cells/well). The following day, complete media was replaced by serum-free DMEM supplemented with 1% (v/v) penicillin/streptomycin. After 24 h, cells were pre-treated with CBD or RGZ for 1 h and stimulated with TGFβ1 (10 ng/ml) for 48 h. At the indicated time points, cell media was collected and assayed for soluble collagen using the Sircol Collagen Assay (Biocolor, County Antrim, United Kingdom) according to the manufacturer’s protocol. NIH3T3 cells were seeded in 24-well plates and after 24 h they were transiently transfected with CAGA-luc plasmid using Roti©-Fect (Carl Roth, Karlsruhe, Germany) following manufacturer´s specifications. The CAGA-luc reporter plasmid contains multimerized Smad-binding elements that bind active Smad2/3 complexes. After stimulation, the luciferase activities were quantified using Dual-Luciferase Assay (Promega, Madison, WI, United States). To correct for transfection efficacy, 100 ng Renilla luciferase (pRL-CMV) was cotransfected.

### Fibroblast scratch assay

Normal human dermal fibroblasts (NHDF) (2 × 10^4^ cells/well) were seeded in 96-well plates (Essen Bioscience, Newark, United Kingdom). Once fibroblast reached confluence, a scratch was made using the 96-pin WoundMaker (Essen Bioscience). Then, cell media was replaced by fresh serum-free DMEM supplemented with 1% (v/v) penicillin/streptomycin. At that point, CBD treatments were added in combination with either TGFβ1 or rhIL-4 (10 ng/ml) to induce cell proliferation. Images were taken every 3 h for 48 h, and data analysed using IncuCyte HD software.

### Western blots

NHDF cells were incubated in low serum conditions (1% FBS) for 24 h. Then cells were pretreated with CBD or Rosiglitazone (RGZ) for 1 h and stimulated with TGFβ1 (10 ng/ml) for 2 h. After treatments, the cells were washed with PBS and proteins extracted in 50 μL of lysis buffer (50 mM Tris–HCl pH 7.5, 150 mM NaCl, 10% glycerol and 1% NP-40) supplemented with 10 mM NaF, 1 mM Na3VO4, 10 *µ* g/ml leupeptine, 1 μg/ml pepstatin and aprotinin, and 1 μL/ml saturated PMSF. Protein concentration was determined by the Bradford assay (Bio-Rad, CA, United States) and 30 μg of proteins were boiled at 95°C in Laemmli buffer and electrophoresed in 10% SDS/PAGE gels. Separated proteins were transferred to PVDF membranes (20 V for 30 min) and blocked in TBS solution containing 0.1% Tween 20 and 5% non-fat dry milk for 1 h at room temperature. Immunodetection of specific proteins was carried out by incubation with primary antibody against pSMAD2 (1:500; #AB3849, Merck Millipore), SMAD2 (1:500; #5339, Cell Signaling, MA, United States) or β -actin (1:10.000; #A5316, Merk, St Louis, MO, United States) overnight at 4°C. After washing membranes, horseradish peroxidase-conjugated secondary antibody was added and detected by chemiluminescence system (GE Healthcare Europe GmbH).

### Animals

Animal work was performed in compliance with the ARRIVE and European Union guidelines and procedures were approved by the Animal Research Ethic Committee of the University of Cordoba and the Andalusian Regional Committee for Animal Experimentation (07/04/2021/044 and 03/11/14/145). Mice were housed under constant conditions of light (12 h light/dark cycle), temperature (20 ± 2 °C) and relative humidity (40–50%), with free access to standard food and water. Handling of animals was performed in compliance with the guidelines of animal care set by the EU guidelines 86/609/EEC. Measures to improve welfare assistance and clinical status as well as endpoint criteria were established to minimize suffering and ensure animal welfare. CBD dosing was chosen based on previous experience using CBD derivatives for treating fibrosis (Del Rio et al., 2018) and literature reporting pharmacological effects of CBD *in vivo* (Rajesh et al., 2010; Cheng et al., 2014; Austrich-Olivares et al., 2022).

### Mice model of skin fibrosis

BALB/c female mice aged 6–8 weeks (Envigo, Valencia, Spain) were housed in groups of nine animals and acclimatized to manipulators for a week before the experiment. Skin fibrosis was induced by daily subcutaneous (s.c.) administration of BLM (50 μg/mice; 100 μL; Mylan, Barcelona, Spain) into the back for 3 weeks. Treatments were administered for 3 weeks in parallel to fibrosis induction by daily i. p. injections of CBD (20 mg/kg; 100 μL) or vehicle (4% DMSO, 6.2% Tween 20 in saline; 100 μL). Control group received s. c. saline instead of BLM and i. p. vehicle. During the protocol, mice were evaluated daily, and weight was monitored weekly. No significant changes in weight or behaviour were observed. Mice were euthanized by cervical dislocation and the back skin was collected. Macroscopic evaluation of internal organs did not reveal any pathological changes.

### Induction of CCl4-induced liver fibrosis

Six-week-old male C57BL6 mice (from Charles Rivers Laboratories; l’Arbresle, France) were acclimated for 2 weeks. When eight-week-old, hepatic fibrosis was induced by intraperitoneal (ip) injection of 1 ml/kg body weight (BW) carbon tetrachloride (CCl4) -vehicle corn oil 1:4-, twice weekly for 2 weeks (Scholten et al., 2015). Pair aged mice received the corresponding vehicle injections. Concurrent with the induction of hepatic fibrosis, mice were daily administered *via* oral gavage with vehicle (sesame oil) or CBD (20 mg/kg) for 2 weeks. CCl4, corn oil and sesame oil were purchased from Sigma-Aldrich.

### Histological evaluation

Mice were euthanized 24 h after the last BLM administration or 72 h after the last dose of CCl4. Tissue samples were collected and fixed for a period of at least 48 h in fresh 4% paraformaldehyde (PFA) in 0.1 M PBS for monitoring progression of inflammation and fibrosis by histochemical analysis. Tissues were processed for histological analysis by formalin fixation. Paraffin-embedded skin and liver sections (5 μm-thick) were stained with Masson’s trichrome technique. Toluidine blue staining was used for the detection of mast cells in the skin. Liver paraffin-embedded tissue sections were stained with Picrosirius Red (PSR) staining following manufacturer’s instructions (Sigma-Aldrich) in order to detected liver collagen. Tenascin C expression, T lymphocyte and macrophage infiltration were detected with anti-TNC (1:100) (MAB3138, R&D Systems, Minneapolis, MN, United States), rat anti-CD3 (1:100) (ab11089, Abcam, Cambridge, United Kingdom), or anti-F4/80 (1:50) (MCA497, Bio Rad, Hercules, CA, United States) primary antibodies overnight at 4°C, respectively. For blocking endogenous mouse IgG and non-specific background, rodent block M (RBM961, Biocare Medical, Concord, CA) was used prior anti-CD3 antibody. Then, the slides were incubated for 1 h at room temperature with the appropriate biotin-conjugated secondary antibodies; goat anti-mouse (21538, Merck-Millipore) for CD3 and goat anti-rat (BP-9400, Vector Laboratories, Burlingame, CA, United States) for TNC and F4/80. Reaction products were detected by avidin-biotin-peroxidase (Vector Laboratories), the color reaction was developed with DAB (3,3′Diaminobenzidine) chromogen (Dako, Santa Clara, CA, United States) and subsequent counterstained with hematoxylin. Samples were analysed with a Leica DM2000 microscope and pictures were taken with a Leica MC190 or Leica DFC420c cameras and analysed using ImageJ software (https://imagej.nih.gov/ij/) for quantification.

### Real-time PCR

Total RNA extraction from mice skin was performed using Qiazol lysis reagent (Qiagen, Hilden, Germany) and purified with RNeasy Lipid Mini Kit (Qiagen). 1 μg of total RNA was retrotranscribed using iScript™ cDNA Synthesis Kit (Bio-Rad). Real-time PCR was performed using the iQTM SYBR Green Supermix (Bio-Rad) in a CFX96 Real-Time PCR Detection System (Bio-Rad). Gene expression was normalized to GAPDH in each sample and expressed using the 2^−ΔΔCt^ method. The oligonucleotide primers sequence used are listed in Table 1.

**TABLE 1:** Primer sequence information.

Primer	Sequence
*mIl6 Fw*	GTA​TGA​ACA​ACG​ATG​ATG​CAC​TTG
*mIl6 Rv*	GTA​TGA​ACA​ACG​ATG​ATG​CAC​TTG
*mIl1β Fw*	CTC​CAC​CTC​AAT​GGA​CAG​AA
*mIl1β Rv*	GCC​GTC​TTT​CAT​TAC​ACA​GG
*mTnc* Fw	CCA​CCA​AGT​TTA​CCA​CAG​ACC​T
*mTnc Rv*	TCC​ACA​GAT​TCA​TAG​ACC​AGG​AG
*mGapdh Fw*	TGG​CAA​AGT​GGA​GAT​TGT​TGC​C
*mGapdh Rv*	AAG​ATG​GTG​ATG​GGC​TTC​CCG

### Statistical analysis

Statistical analyses were performed using Prism software (GraphPad Prism version 8.00, GraphPad Software, La Jolla, California, United States, https://www.graphpad.com/). *In vivo* data are expressed as the mean ± SEM. Unpaired two-tailed student t test or one-way analysis of variance (ANOVA) followed by Tukey’s post-hoc test for parametric analysis or Kruskal–Wallis post-hoc test for non-parametric analysis were used to determine the statistical significance. The level of significance was set at *p* < 0.05. Statistical details of each experiment can be found in the figures and the respective figure legends.

## Results

### Cannabidiol limits the profibrotic response *in vitro*

**FIGURE 1:**
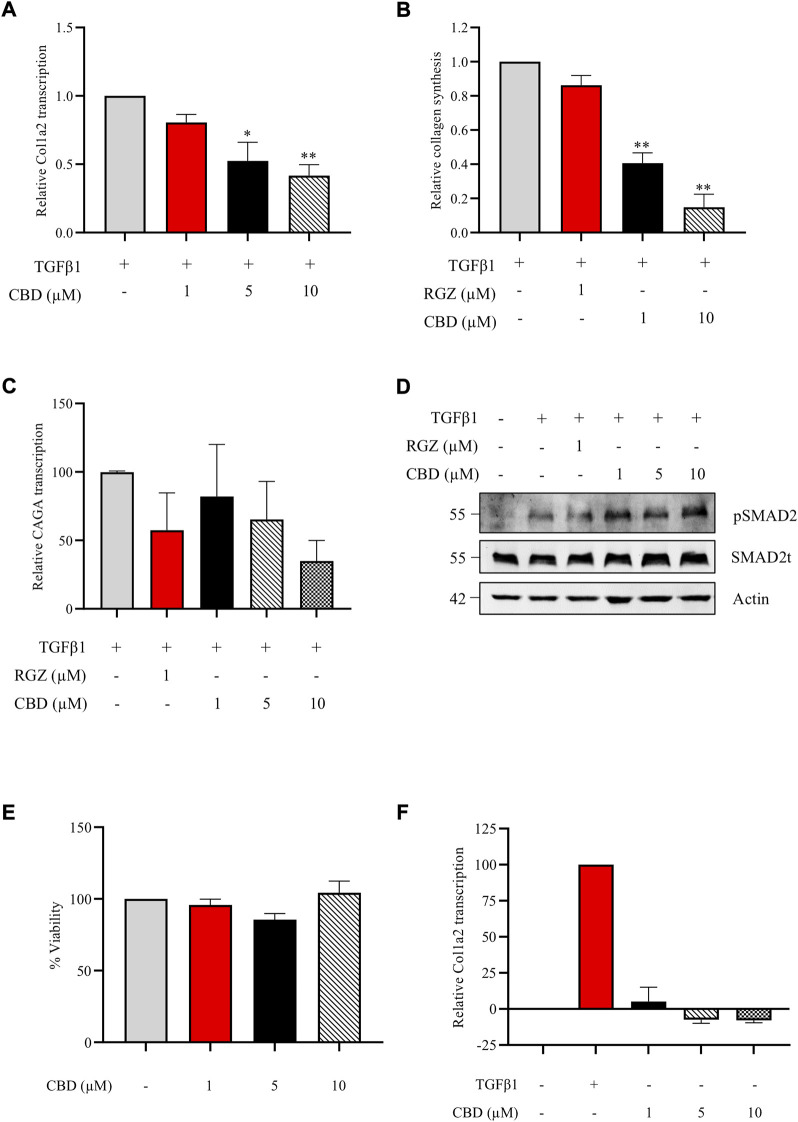
CBD inhibits collagen transcription and synthesis *in vitro* without altering SMAD2 pathway. **(A)** Col1a2 transcriptional activity in NIH-3T3 cells pretreated with CBD for 1 h and stimulated with TGFβ1 for the following 24 h (n = 4) **(B)** Effect of CBD pretreatment on soluble collagen release by human dermal fibroblast using the Sircol Assay. Fibroblasts were pretreated with CBD for 1 h and stimulated with TGFβ1 for 48 h and collagen was measured in the culture media (n = 3). **(C)** Effect of CBD on SMAD-dependent transcriptional activity. NIH-3T3 cells were transfected with the CAGA-Luc plasmid, preincubated with the indicated concentrations of CBD for 1 h and stimulated with TGFβ1 (10 ng/ml) for 6 h. Then, cells were lysed for luciferase activity (*n* = 3). Data are expressed as mean ± S.D. relative to control cells. *p < 0.05; **p < 0.01; ***p < 0.001 vs TGFβ1-treated cells. **(D)** CBD effect on SMAD2 phosphorylation. NHDF cells were incubated in low serum conditions (1% FBS) for 24 h. Then cells were pretreated with CBD or Rosiglitazone (RGZ) for 1 h and stimulated with TGFβ 1 (10 ng/ml) for 2 h. **(E)** Viability of NIH3T3 cells treated with different concentrations of CBD after 24 h expressed as percentage taking control as 100% (*n* = 3). **(F)** Effect of CBD treatment on Col1a2 transcriptional activity in NIH-3T3 cells (*n* = 3).

Accumulating evidence suggest that CBD, the main non psychotropic cannabinoid present in *Cannabis sativa*, exerts a protective role in fibrotic conditions. We first investigated the *in vitro* effect of CBD against the profibrotic activity induced by TGFβ1, the primary factor driving fibrosis. NIH-3T3 cells transiently transfected with the Col1A2-luc plasmid were pretreated with CBD at different non-cytotoxic concentrations. CBD treatment resulted in a significant inhibition of TGFβ1 stimulation on Col1A2 transcription in a concentration-dependent manner (Figure 1A). However, no effects were found when NIH3T3 cells were treated with CBD alone (Figure 1F). Next, we studied the resulting collagen synthesis using NHDFs stimulated with TGFβ1 for 48 h. NHDFs preincubated with increasing concentrations of CBD from 1 h prior to the addition of TGFβ1 showed a significant reduction in collagen release (Figure 1B). Also, we tested the capacity of CBD to interfere with upstream or downstream TGFβ signaling pathways in comparison with Rosiglitazone (RGZ), a PPARγ agonist. We found that both compounds were able to inhibit the transcriptional activity driven by SMAD proteins in CAGA-Luc transfected NIH-3T3 cells (Figure 1C). However, neither CBD nor RGZ inhibited TGFβ-induced SMAD2 phosphorylation (Figure 1D). CBD has been shown to activate PPARγ and our results are consistent with the view that PPARγ agonists inhibit the expression of several TGFβ-activated genes by acting at the transcriptional level.

**FIGURE 2:**
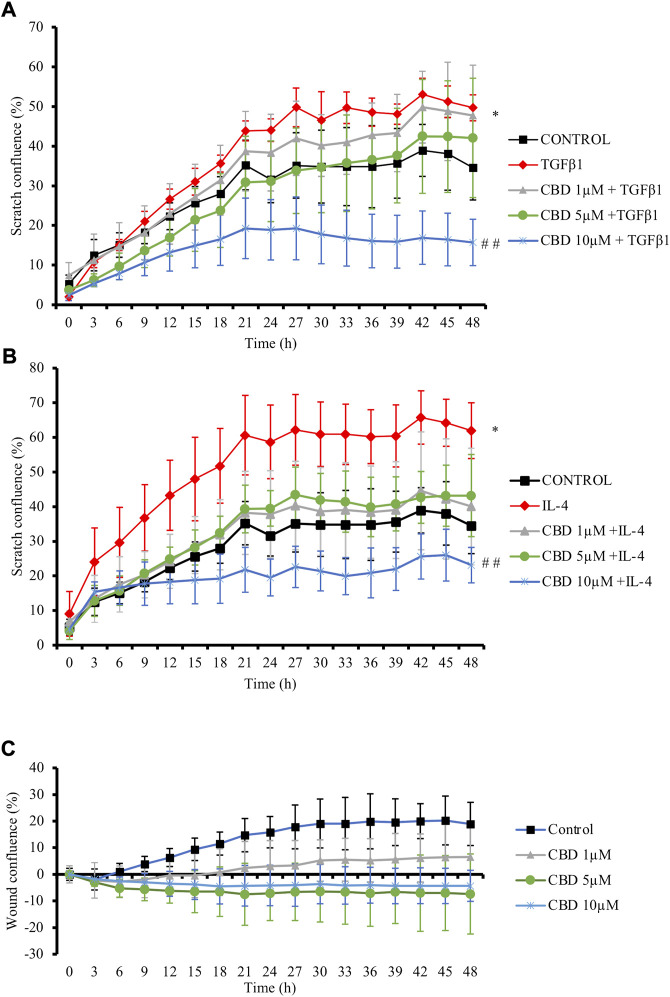
CBD reduces *in vitro* fibroblast migration. NHDF monolayers were scratched and treated with TGFβ1 **(A)** or IL-4 **(B)** in the presence of CBD or with CBD alone **(C)**. Results are expressed as percentage of closure (confluence) ± SD (n = 3). *p < 0.05 *versus* control; ^# #^ *p* < 0.01 vs TGFβ1-treated cells.

Increased speed of resident cells migration relative to the normal tissues is also promoted by profibrotic cytokines and plays a crucial role during fibrogenesis (Kai et al., 2016). Then, the effects of CBD on fibroblast migration were evaluated in NHDFs cells monolayer, which were scratched and treated with CBD in parallel to TGFβ1 or IL-4 for 48 h. TGFβ1 (Figure 2A) and IL-4 (Figure 2B) fostered cell confluence in the scratch while treatment with CBD at the highest non-cytotoxic concentration significantly reduced fibroblast migration by 35% in both conditions. Additionally, CBD alone did not exert any effect on scratch confluence (Figure 2C).

### Effect of cannabidiol on skin fibrosis induced by BLM administration

**FIGURE 3:**
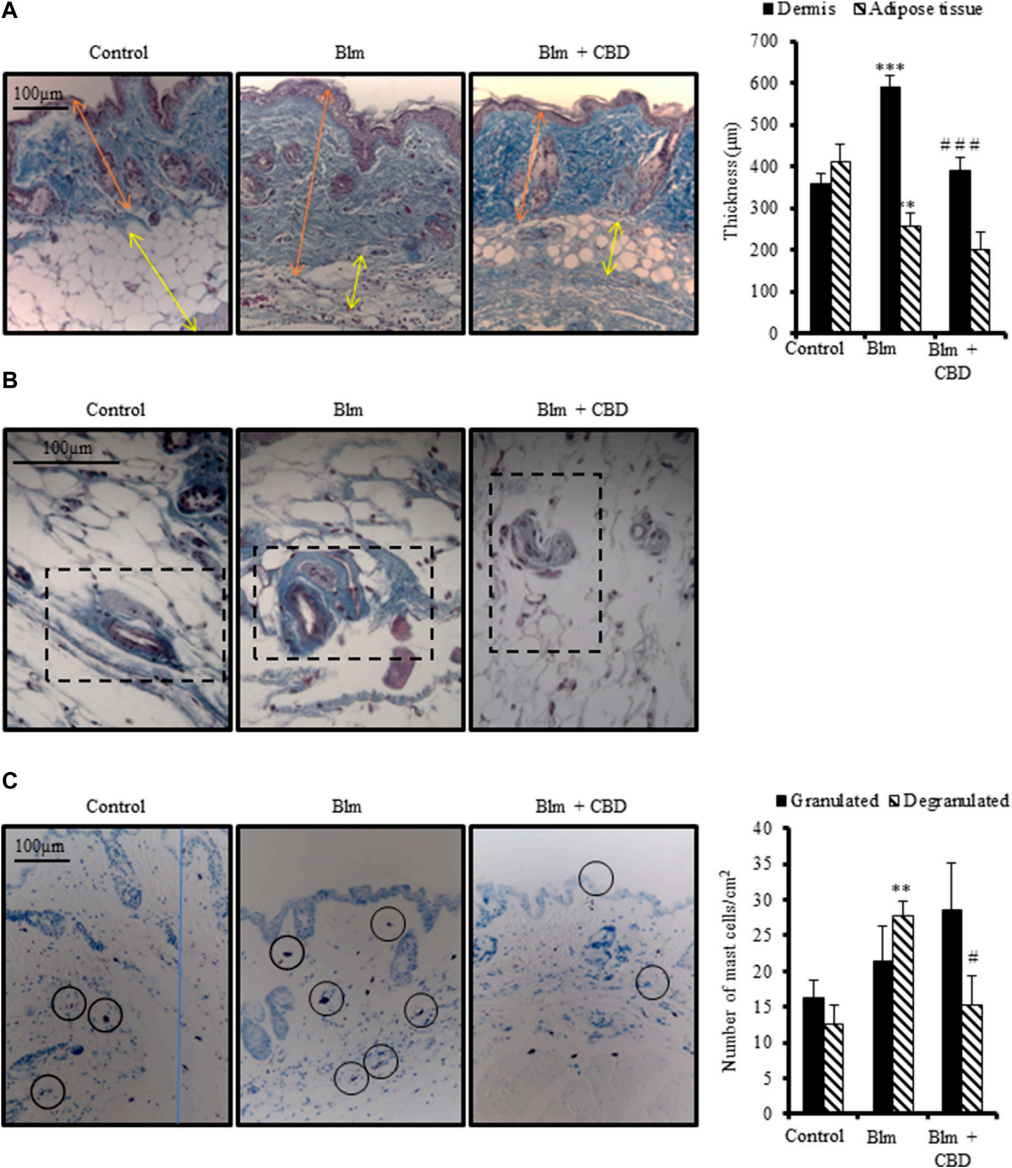
Treatment with CBD reduced skin fibrosis development in a mouse model of systemic sclerosis. Skin fibrosis was induced by daily subcutaneous administration of BLM for 3 weeks and mice were treated in parallel with CBD or vehicle i. p. injections (n = 9 mice per group). **(A)** Representative images of Masson’s trichrome stained skin sections and quantification of dermal and subcutaneous adipose layers thickness. **(B)** Masson’s trichrome staining showing collagen around skin blood vessels. **(C)** Representative images of toluidine blue stained skin sections showing mast cell degranulation and their corresponding quantification. Data represent the mean ± SEM. *p < 0.05; ***p < 0.001 vs control mice. ^#^ *p* < 0.05; ^# #^ *p* < 0.01 vs BLM-treated mice.

Repeated subcutaneous administration of BLM is the most common model to study skin fibrosis. As expected, female BALB/c mice injected with BLM for 3 weeks developed skin thickening accompanied by a reduction of subcutaneous adipose layer. Treatment with CBD for 3 weeks during fibrosis induction significantly prevented dermal thickness secondary to collagen deposition (Figure 3A). However, CBD was not able to avoid significantly the reduction of subcutaneous adipose tissue. In addition, BLM induced vascular lesions manifested by the thickening of the vascular wall. CBD-treated mice showed reduced collagenic bundles around blood vessels to levels comparable to the control group (Figure 3B).

**FIGURE 4:**
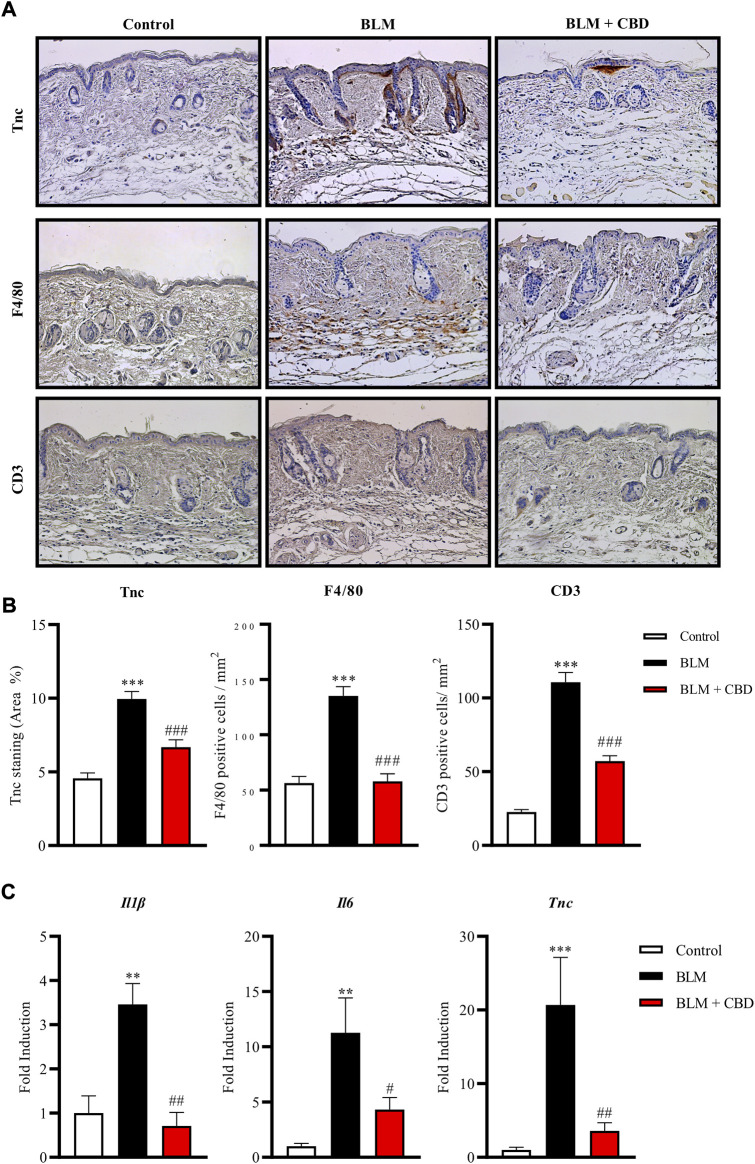
Treatment with CBD reduced skin fibrosis and inflammation in a mouse model of systemic sclerosis. **(A)** Representative images of Tnc, F4/80 and CD3 immunohistochemistry. **(B)** Quantifications of Tnc, F4/80 and CD3 expression were performed with Image J software. Values are expressed as mean ± SEM. ***p < 0.001 vs control mice. ^# # #^ *p* < 0.001 vs BLM-treated mice. **(C)** Gene expression of inflammatory and fibrotic markers including *Il-6*, *Il-1β* and *Tnc* was significantly downregulated in CBD-treated mice compared with BLM mice. Data represent the mean ± SEM (n = four to six animals per group). **p < 0.01; ***p < 0.001 vs control mice. ^#^ *p* < 0.05; ^# #^ *p* < 0.01 vs BLM-treated mice.

It is well known that the number of mast cells increase during the development of fibrosis in several tissues and early fibrogenesis associates with marked cell degranulation (Yamamoto and Nishioka, 2005). Accordingly, the lesioned skin of BLM treated mice presented a higher number of degranulated mast cells. CBD administration led to one-fold decrease in the number of degranulated mast cells in the skin (Figure 3C). Next, we studied the levels of Tenascin C (Tnc) in the skin. Tnc is involved in modulating the extracellular matrix (ECM) composition and is known to stimulate the profibrotic response and upregulate the expression of type I collagen by fibroblasts (Bhattacharyya et al., 2016). Local BLM administration in the skin elevated Tnc stained area while CBD treatment significantly diminished this Tnc upregulation (Figures 4A,B). In BLM-challenged mice a significant increase in the recruitment of inflammatory cells was observed. Mice treated with CBD exhibited a significant reduction of macrophages and T cells infiltration in the skin (Figures 4A,B). Consequently, the expression of cytokines associated with inflammation and fibrosis was analysed. As expected, BLM promoted Il1β, Il6 and Tnc expression in the skin, and intraperitoneal treatment with CBD prevented the proinflammatory boost induced by BLM administration (Figure 4C).

### Effect of cannabidiol on CCl4-induced liver fibrosis

**FIGURE 5:**
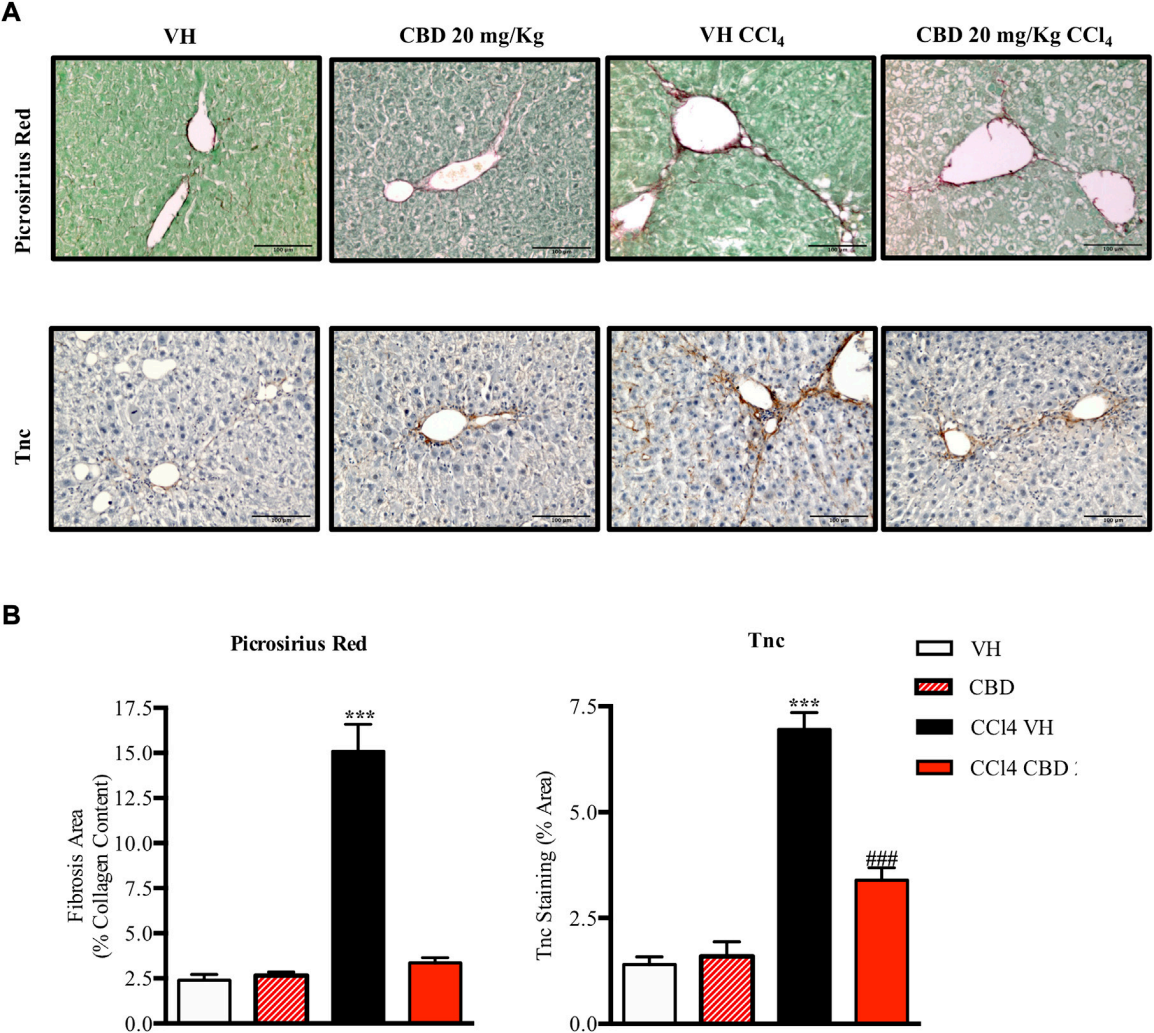
CBD reduces liver fibrosis induced by CCl4. **(A)** Representative images of collagen staining in the liver using picrosirius red dye (upper panel) or immunostaining with Tnc (bottom panel) from control, CBD, CCl4-vehicle, and CCl4 + CBD mice. Scale bars represent 100 and 50 μm, respectively. **(B)** Quantification of positive collagen content (left panel) and Tnc staining (right panel), expressed as a percentage of the total liver area. Values are expressed as mean ± SEM (n = 6 animals per group). ***p < 0.001 vs. control group; ^###^ *p* < 0.001 vs. CCl4 group (ANOVA followed by Tukey’s test).

**FIGURE 6:**
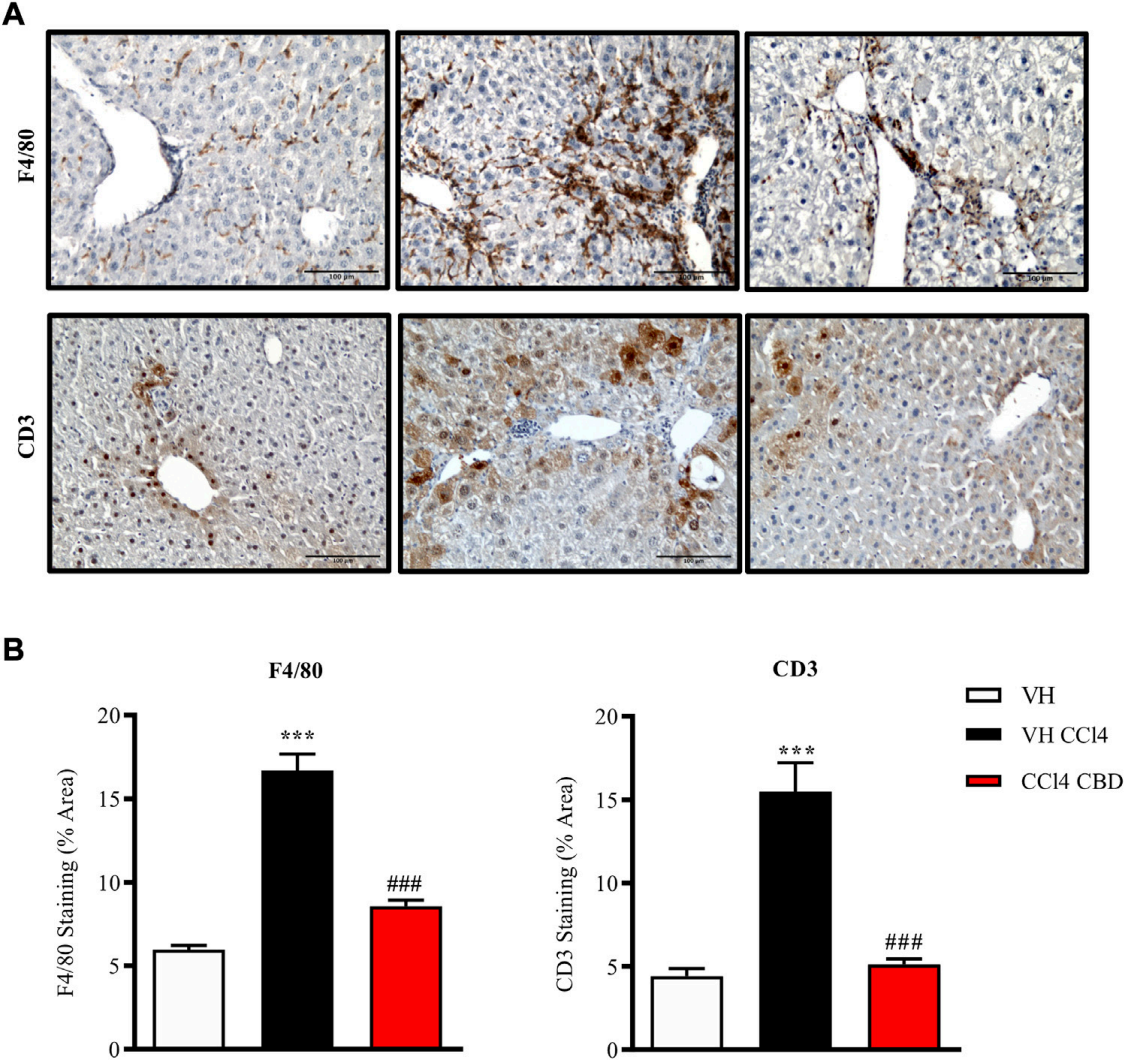
CBD reduces hepatic inflammation induced by CCl4. **(A)** Representative images of immunostaining for F4/80 (macrophage-specific marker; upper panel) and CD3 (lymphocyte-specific marker; bottom panel) from control, CCl4-vehicle, and CCl4 + CBD mice. Scale bars represent 100 μm. **(B)** Quantification of (percentage of total liver area) for F4/80 and CD3 (left and right panel respectively). Values are expressed as mean ± SEM (n = 6 animals per group). ***p < 0.001 vs. control group; ^###^ *p* < 0.001 vs. CCl4 group (ANOVA followed by Tukey’s test).

Next, we were also interested to study the antifibrotic and antiinflammatory effects of orally delivered CBD in another model of fibrosis. Eight-week-old C57Bl6J mice were randomized to healthy control group, CCl4-treated group in combination with vehicle (CCl4-vehicle) and CCl4-treated group in combination with 20 mg/kg of CBD. In addition, a group of mice treated with CBD alone was also included for reference purposes. As reported previously, PSR staining revealed that CCl4 treatment significantly induced accumulation of collagen in the liver compared to the healthy group, and CBD greatly reduced the fibrotic liver area compared to CCl4-vehicle mice, reaching levels close to the control group (Figure 5). Furthermore, CCl4 treatment also increased the protein level of Tnc, which is also an early fibrotic marker in liver (Kasprzycka et al., 2015), while CBD co-treatment led to a 2-fold reduction of protein levels of Tnc compared to CCl4-vehicle mice. Importantly, CBD in the absence of CCl4 did not induce fibrosis. In hepatocytes, cytochrome P450 proteins (CYP2E1) metabolize endogenous substrates as well as xenobiotic compounds, including carbon tetrachloride. In this sense, hepatic transformation of CCl4 produces trichloromethyl radicals, which trigger several free radical reactions and contribute to the induction of an inflammatory response. Immunostaining for CD3 showed that CCl4 induces a significant (3.7-fold) increase in CD3^+^ T lymphocyte infiltration into the liver compared to control mice, which was reduced in animals treated with CBD (2.9 fold-increase; Figure 6). Similarly, liver sections in CCl4-vehicle mice had 2.8-fold higher infiltration of macrophages than healthy animals, as seen by F4/80 staining (Figure 6), and CBD induced a significant reduction (1.9-fold) in the magnitude of liver infiltration of F4/80 + cells compared to CCl4-vehicle group.

## Discussion

Different reports have evidenced the antifibrotic effects of CBD *in vitro* and in various preclinical animal models of fibrotic diseases (Rajesh et al., 2010; Lee et al., 2016; De Ternay et al., 2019; Vuolo et al., 2019). To the best of our knowledge the antifibrotic effects of CBD on skin and liver fibrosis have not been studied previously. Herein, we have shown that treatment with CBD not only alleviated organ fibrosis but also attenuated inflammation, which is a major driving force for fibrosis development. We also found that CBD inhibits collagen gene transcription and synthesis and fibroblast migration *in vitro*.

The antifibrotic effects of CBD are still elusive and could be attributed to different signaling pathways. CBD weakly binds to the orthosteric binding sites of CB_1_ (Ki values in the micromolar range) and CB_2_ (Ki in the high nanomolar range) receptors (Rosenthaler et al., 2014). However, CBD is a negative allosteric modulator (NAM) for CB_1_ receptor (Laprairie et al., 2015) and a CB_2_ inverse agonist (Thomas et al., 2007; Martinez-Pinilla et al., 2017). There is evidence that CB_1_ receptor activation is profibrotic in different fibrotic conditions (reviewed by (Rio et al., 2018). For instance, CB_1_ deficient mice were protected against experimental fibrosis and the activation of CB_1_ exacerbated mouse experimental fibrosis induced by BLM (Marquart et al., 2010). Therefore, CBD could exert its antifibrotic activity, at least in part, by acting as a NAM on CB_1_ receptor.

CBD’s multimodal pharmacologic profile further includes other receptors such as PPARγ that may explain its antifibrotic and antiinflammatory effects (O'Sullivan et al., 2009). PPAR*γ* is a negative regulator of the inflammatory response, inhibits collagen synthesis and blunts fibrogenesis in a wide variety of organs (Dantas et al., 2015; Li et al., 2021). Skin fibrosis is associated with a progressive loss of PPAR*γ* expression and activation of PPARγ using rosiglitazone reduced inflammation and dermal fibrosis (Wu et al., 2009). Activation of PPARγ by rosiglitazone also ameliorated bile duct ligation-induced liver fibrosis (Wei et al., 2019). Moreover, PPARγ viral overexpression has been reported to reduce CCl4-induced liver fibrosis in rats (Wang et al., 2011).

It is well known that BLM and CCl4 can induce cell damage and organ fibrosis through inflammatory processes, attributed in part to their free radical-promoting ability. CBD beneficial effects can be also explained in part by its potent antioxidant properties. It is plausible that the antioxidant activity of CBD can reduce the generation of the reactive oxygen species (ROS) in these mouse models and therefore limit the pathological manifestations. Accordinlgy, classical antioxidants, like phenolic compounds, were effective in preventing tissue inflammation and fibrosis (Pan et al., 2018; Shariati et al., 2019). Unlike other cannabinoids, CBD contains two phenolic groups. Direct antioxidant properties of CBD may be related to the location and surroundings of the hydroxyl groups in the phenolic ring (Atalay et al., 2019). Two main mechanisms can explain the protective role of CBD as an antioxidant: electron (the antioxidant compound give an electron to the radical) and hydrogen (the free radical removes a hydrogen atom from the antioxidant) abstraction (Silva, 2017). CBD also modulates antioxidant gene expression by inducing the activation of Nrf2, the master regulator of the antioxidant response (Singer et al., 2015). Recent report indicated that CBD is a weak inducer of Keap1/Nrf2 activation but a potent Bach1 inhibitor (Casares et al., 2020). Bach1 is a transcriptional repressor of Nrf2 and its inactivation by CBD mediated the expression of heme oxygenase 1 (HMOX1), an essential enzyme in oxidative degradation of heme group (Casares et al., 2020). Interestingly, HMOX-1 is thought to provide antinflammatory and antibrotic activity in different preclinical models (Barikbin et al., 2012; Wang et al., 2013).

We have studied the effect of intraperitoneal and oral CBD, administered since the initial induction of fibrosis, and therefore acting simultaneously to the development of the disease, which includes CD3^+^ and F4/80^+^ immune cell infiltration prior to collagen deposition. CBD antioxidant profile, together with its actions on PPARγ, Adenosine A_2A_ receptor and TRPV1 (Carrier et al., 2006) (Feng et al., 2017), are likely to be targeting the major pro-inflammatory and pro-oxidant signalling pathways involved in the initial stages of tissue injury leading to abnormal remodelling and, subsequently, fibrosis (reviewed by (Sunda and Arowolo, 2020)). The same was observed for Δ^9^-THCA, which only showed antifibrotic effect in the liver when the treatment started at the same time of CCl_4_ challenge (Carmona-Hidalgo et al., 2021). However, although we have not tested the effect of CBD on preestablished fibrosis, a therapeutic effect can not be discarded given that CBD directly inhibited fibroblast migration and collagen transcription *in vitro*.

CBD metabolism has been demonstrated *in vitro* and *in vivo* (Martin et al., 1976; Brown, 1990). The route of administration considerably affects CBD pharmacokinetics. Oral bioavailability of CBD is low across species, known to be approximately 6% in humans (Nakano et al., 2019). We have demonstrated that both intraperitoneal and oral treatments are able to reduce inflammation and fibrosis. Oral administration usually delays serum peak concentration and sometimes show a second peak due to enterohepatic circulation (Hlozek et al., 2017). In the liver, CBD is metabolized by CYP2C19 and CYP3A4 isozymes and undergoes hydroxylation at multiple sites and further oxidations (Anderson and Chan, 2016). About a hundred CBD metabolites have been identified. Main CBD metabolites are hydroxylated 7-COOH derivatives of CBD but little is known about their pharmacological activity. *In vitro* biological activities of CBD metabolites include antiangiogenic, anticancer and antiinflammatory properties (Ujvary and Hanus, 2016). In the event of CBD metabolites reaching a pharmacologically relevant concentration, direct or indirect contribution to the observed therapeutic effect of CBD *in vivo* can not be excluded.

Altough we have found that CBD alone does not induce liver fibrosis, a major concern of CBD use is the risk of hepatotoxicity. Acute oral administration of a concentrated CBD-enriched extract was reported to produce hepatotoxicity indicated by marked increases in serum ALT, AST, and total bilirubin. However, the concentration used (2,460 mg/kg) is not therapeutically applicable (Ewing et al., 2019). In the same study, subacute 2-week administration of the extract revealed no measurable toxicological responses associated with liver injury in mice orally gavaged with CBD up to 184.5 mg/kg (Ewing et al., 2019). Several studies have reported that pure CBD can be hepatoprotective in mice (Magen et al., 2009; Avraham et al., 2011; Wang et al., 2017). The most common CBD therapeutic dose used for seizure disorders is 20 mg/kg/day. Human studies addressing potential CBD hepatotoxicity events of Epidiolex^®^ included patients concomitantly taking other medications, such as valproic acid which is known for its hepatotoxicity. Therefore, whether CBD is endowed with adverse hepatic effects is unclear (Devinsky et al., 2016; Devinsky et al., 2017), and, overall, human studies indicate limited hepatic effects upon continued use of CBD (Stohs and Ray, 2020).

## Conclusion

We have shown that both intraperitoneal and oral administration of CBD exerts potent anti-inflammatory and antifibrotic activities *in vivo.* Moreover, CBD blunted the effects of fibrogenic stimuli on cultured fibroblast. We have shown for the first time CBD efficacy in reducing BLM-induced dermal fibrosis and CCl4-induced hepatic fibrosis. Given the broad spectrum of CBD targets, *in vivo* effects might be mediated by a plethora of molecular mechanisms, directly or through its metabolites. Further studies are needed for dissecting the exact contribution of each mechanism involved.

## Data Availability

The raw data supporting the conclusions of this article will be made available by the authors, without undue reservation.
